# Gaining a Better Understanding of the Types of Organizational Culture to Manage Suffering at Work

**DOI:** 10.3389/fpsyg.2021.782488

**Published:** 2021-11-22

**Authors:** Jordi Assens-Serra, Maria Boada-Cuerva, María-José Serrano-Fernández, Esteban Agulló-Tomás

**Affiliations:** ^1^Department of Strategy, Leadership and People, EADA Business School, Barcelona, Spain; ^2^Human Factor, Organizations and Markets (FHOM), Faculty of Business and Economics, Universitat Rovira i Virgili (URV), Tarragona, Spain; ^3^Faculty of Psychology, Universidad de Oviedo, Oviedo, Spain

**Keywords:** organizational culture, suffering at work, job (un)satisfaction, intrinsic variables, extrinsic variables, clan culture, market culture, hierarchy culture

## Abstract

Organizational culture is a central concept in research due to its importance in organizational functioning and suffering of employees. To better manage suffering, it is necessary to better understand the intrinsic characteristics of each type of culture and also its relationships with the environment. In this study, we used the multiple regression analysis to analyze the capacity of eight environment variables, five business strategies, and eight organizational competencies to predict the presence of Clan, Market, and Hierarchy cultures (Cameron and Quinn, 1999) in a subsample of Spanish managers (*n*_1_ = 362) and a subsample of Peruvian managers (*n*_2_ = 1,317). Contrary to what most of the literature suggests, we found almost no relationship between the environmental variables and the culture types. Strategy and competencies, in contrast, do have a significant predictive capacity, showing 9 links with the Clan culture, 7 with the Hierarchy culture, and 10 with the Market culture. In conclusion, this study has found the important characteristics of the types of organizational culture that could be useful to better manage the suffering of employees.

## Introduction

**FIGURE 1:**
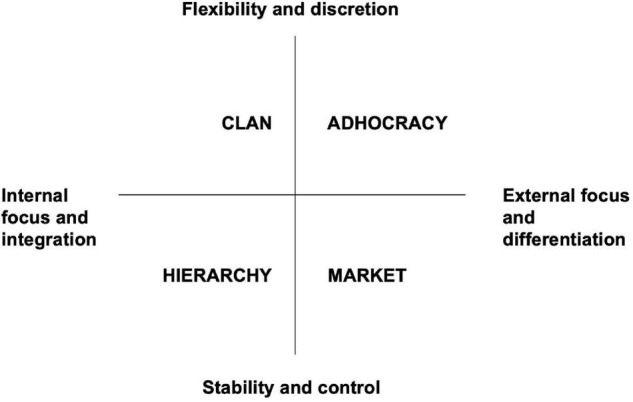
The culture model as suggested by Cameron and Quinn (1999, 2006).

Organizational culture is a central concept in research due to its importance in organizational functioning (Giorgi et al., 2015) and suffering of employees (Gill, 2019). According to the study by Schein (2010), the organizational culture is a pattern of basic values and presuppositions that are shared and learned by a group while resolving the problems of external adaptation and internal integration. A well-established framework for studying culture is the model suggested by Cameron and Quinn (1999) which defines four culture archetypes, namely, Clan, Adhocracy, Market, and Hierarchy. Each culture represents a different set of values and presuppositions. All organizations have all four types but in different proportions. This is a typological model because it aims to identify archetypes using different effectiveness criteria. As shown in Figure 1, the cultures are represented in four quadrants and ordered into two dimensions. The vertical dimension moves from flexibility, discretion, and dynamism to stability, order, and control, while the horizontal dimension moves from internal orientation and integration to external orientation and differentiation.

Research suggests that each culture has a different capacity to avoid suffering and create well-being in employees. The Clan culture is similar to an extended family. It is a very personal place characterized by mentoring, teamwork, participation, and trust (Cameron and Quinn, 2006). Employees in organizations with Clan culture are more satisfied with their jobs and have more behaviors aimed at benefiting peers and companies (Lopez-Martin and Topa, 2019). Clan culture improves the satisfaction of respect needs (Zavyalova and Kucherov, 2010) and is also negatively associated with conflict (McClure, 2010). Thus, there are different characteristics in Clan culture that could have an effect of reducing suffering. The Market culture is very results-oriented. People are very competitive, and market leadership is a key (Cameron and Quinn, 2006). Workers in these organizations perceive their health as worse than average (Lopez-Martin and Topa, 2019) despite this culture creates the conditions for the satisfaction of self-affirmation needs (Zavyalova and Kucherov, 2010). Therefore, Market culture has characteristics that could increase suffering in employees. The Hierarchy culture is characterized by stability, formal rules and policies, strong focus on internal processes, efficiency, control, smooth operations, and low-cost production (Cameron and Quinn, 2006). It is associated with employee demotivation (Adler and Borys, 1996; Cameron and Quinn, 2006) and conflict (McClure, 2010). It also has a significant risk of creating human dysfunctions that harm customer involvement (Naor et al., 2008) and market orientation (Gebhardt et al., 2006). Nevertheless, if the organizational culture is oriented toward rules, it favors the satisfaction of cooperation and safety needs (Zavyalova and Kucherov, 2010), and employees report a better health status (Lopez-Martin and Topa, 2019). Therefore, the Hierarchy culture appears to have characteristics that could both increase and decrease suffering at work. In conclusion, there is a link between organizational culture and suffering at work, but, to better manage suffering, there is the need to understand, in greater depth, the extrinsic and intrinsic characteristics of each organizational culture.

There is widespread agreement in academia that, to survive, the organizational culture needs to be adapted to the environment (Denison et al., 2003; Schein, 2010; Bayraktar et al., 2017; De Clercq et al., 2018). The environment can be measured using extrinsic variables such as market turbulence, technological turbulence, and competitive intensity, which can all move from a stable to a turbulent, aggressive state. Quinn and Cameron (1983) and Schein (2010) noted that the literature on the contingent model of organizational adaptation affirms that companies in changing environments need to have organic and adaptable cultures and structures. Nevertheless, at present, the relationships between the culture and the environment remain unclear.

Another area that needs more research is the internal configuration of each culture, which can be measured using intrinsic variables. These variables are mainly the business strategy and the organizational competencies, which are both developed by founders and leaders of an organization (Berson et al., 2008; O’Reilly et al., 2014) with the aim of making the organization more competitive. What leaders pay attention to, reward, monitor, and talk about focuses the attention and efforts of their followers (Schein, 2010; Braunscheidel and Suresh, 2018). These intrinsic variables have the presuppositions and values of a company embedded within them and make the culture robust (Schein, 2010; Denison et al., 2014; Zohar and Polachek, 2014).

The business strategy of a company refers to the decisions taken by its leaders to achieve a competitive advantage in its market. Mintzberg and Quinn (1995) said that there must be a plan that defines the action to be taken in different situations for the purposes of achieving defined objectives. The business strategy must be proactively articulated with a pattern of actions and behaviors that are aligned with company values and reflect the ideology and philosophy of a company. It must also position the organization in a context and in relation to its environment and stakeholders (Prajogo, 2016). The strategy is, therefore, one of the most important decisions made by the founders and leaders, who will aim to align the culture with it (Slater et al., 2010; Madero Gómez and Barboza, 2015; Marshall, 2018; Barros and Fischmann, 2020).

Organizational competencies, in contrast, are certain capacities the company has that combine knowledge and skills and are necessary for obtaining a competitive advantage (King et al., 2001). They include market orientation, competitor orientation, and type of innovation. Barney (1991) referred to them as a set of internal knowledge-based resources and capabilities. He also noted that they have to be valuable, rare, inimitable, and lack substitutes. Some researchers have empirically shown several relationships between cultures, strategies, and competencies (Olson et al., 2005; Slater et al., 2010), but the intrinsic characteristics of each culture are still not clear. In summary, which cultures are better adapted to each environment and the characteristics of each culture are not well-documented in the literature, which is a matter of concern for practitioners and academics alike.

The goal of this study was to determine which extrinsic and intrinsic variables can predict the presence of the Clan, Market, and Hierarchy organizational cultures (Cameron and Quinn, 1999). Links between each culture and the extrinsic variables could help us understand which cultures are better adapted to each environment. Links with the intrinsic variables could give us information about the business strategies and organizational competencies that characterize each culture. A better understanding of the variables of each organizational culture could be useful to mitigate the suffering of employees. We also compared the results for a Spanish and a Peruvian sample to determine the differences that could enrich our understanding of the cultures. We analyzed some hypotheses from the scientific literature, but the study was also exploratory and aimed to find new links between each culture and the extrinsic and intrinsic variables.

Related to the extrinsic variables, Quinn and Cameron (1983) and Schein (2010) noted that the literature on the contingent model of organizational adaptation states that companies in changing environments need to have organic and adaptable cultures. More specifically, Cameron et al. (2006) used their model to explain how the culture adapts to the environment. In a study that analyzed responses from over 80,000 professionals from more than 3,000 companies of the United States, they found that the Market culture, because it is externally oriented, is the most capable of surviving in an ever-changing environment. Meanwhile, the Clan and Hierarchy cultures, which are internally oriented, are better adapted to stable environments. Accordingly, the following hypotheses are proposed:

Hypothesis C1: the presence of the Clan culture can be predicted by a state of stability in the extrinsic variables.

Hypothesis M1: the presence of the Market culture can be predicted by a state of turbulence in the extrinsic variables.

Hypothesis H1: the presence of the Hierarchy culture can be predicted by a state of stability in the extrinsic variables.

Related to the business strategy, Slater et al. (2010), in an empirical study carried out mainly with marketing managers in the United States, determined some links between the cultures and the strategy. First, competitiveness in the Clan culture is based on very competent, motivated human capital that differentiates the company from competitors. Second, the Market culture focuses on getting results. To do this, it analyzes the market leaders and tries to aggressively compete with them. Third, the Hierarchy culture competes with excellent systems and processes by trying to lower the costs and positioning their offer according to the competitors. These links were also suggested by Cameron et al. (2006). Based on the earlier discussion, the following hypotheses are proposed:

Hypothesis C2: the presence of the Clan culture can be predicted by the Differentiated defender strategy.

Hypothesis M2: the presence of the Market culture can be predicted by the Analyzer defender strategy.

Hypothesis H2: the presence of the Hierarchy culture can be predicted by the Low-cost defender strategy.

Hypothesis H3: the presence of the Hierarchy culture can be predicted by the Analyzer defender strategy.

Related to the intrinsic variables, the same study (Slater et al., 2010) also suggested some links between the cultures and different organizational competencies. First, the Clan culture can be very close to the market and deliver an excellent service thanks to motivated human capital. Its organization is also adaptable and flexible. Second, the Market culture is externally oriented and, to get results, focuses on clients and competitors. Third, the Hierarchy culture uses its systems and processes to provide a better service and better prices than its competitors. It, therefore, continuously needs to benchmark its position. Again, these links were previously suggested by Cameron et al. (2006). In light of these discussions, the following hypotheses have been postulated:

Hypothesis C3: the presence of the Clan culture can be predicted by Market orientation.

Hypothesis C4: the presence of the Clan culture can be predicted by the Speed of organizational change.

Hypothesis M3: the presence of the Market culture can be predicted by Market orientation.

Hypothesis M4: the presence of the Market culture can be predicted by Competitor orientation.

Hypothesis H4: the presence of the Hierarchy culture can be predicted by Competitor orientation.

## Materials and Methods

### Participants

A total of 1,679 managers participated in this study. This study has two different subsamples: one Spanish (*n*_1_ = 362; 69.9% men and 30.1% women; average age = 42.2 years) and one Peruvian (*n*_2_ = 1,317; 67.5% men and 32.5% women; average age = 35.3 years). Most of the study participants have university studies, and they work in a variety of industries and company sizes.

### Measures

#### Organizational Culture

We assessed culture using the Organizational Culture Assessment Instrument (OCAI; Cameron et al., 2006). The questionnaire was translated and adapted into Spanish (Assens-Serra, 2018) using the exploratory factor analysis (EFA) with a Spanish sample (*n*_1_ = 246) and the confirmatory factor analysis (CFA) with a Latin-American sample (*n*_2_ = 510). The result reduced the four-factor internal structure to a three-factor structure that retains the Clan, Market, and Hierarchy factors (i.e., reducing the number of items in each from six to four) but completely excludes the Adhocracy factor. The study gave rise to a three-factor instrument in Spanish called OCAI-12. CFA shows acceptable indicators (TLI = 0.93, CFI = 0.94, RMSEA = 0.07). Reliabilities are also good (α = 0.74 for Clan, α = 0.79 for Market, and α = 0.71 for Hierarchy). Due to that our study independently analyzes the Clan, the Market, and the Hierarchy cultures, the need of excluding the *ad hoc* type does not influence the quality of our results. There is another study in a different context in which the researcher could not retain a factor (i.e., the Market culture) and also used a reduced OCAI (Karakasnaki et al., 2019). Our study represents the largest activity at present for adapting the OCAI (Cameron and Quinn, 2006) into Spanish, with a Likert scale and using CFA. It shows the difficulty in adapting this scale from English into Spanish. Despite the rigor of the translation method, the different meanings of the words and concepts make it difficult the construct equivalence, and it was necessary to reduce the original OCAI to the point of completely discarding the *ad hoc* factor. Previous translations of the OCAI into Spanish only used EFA and found conflicting results. For example, Núñez et al. (2015) retained the four factors in a study with Mexican companies, but Cerpa-Noya (2018), with a sample of workers in Metropolitan Lima, retained only two factors.

The Clan factor measures the assumption that the company will succeed based on its human capital (sample item: “The management style in the organization is characterized by teamwork, consensus, and participation”). The Market factor measures the assumption that there is a need to compete aggressively to get business results (sample item: “The organization is very results-oriented. A major concern is with getting the job done. People are very competitive and achievement-oriented”). The Hierarchy factor measures the assumption that success comes with stable, predictable, and efficient formal rules and policies (sample item: “The management style in the organization is characterized by the security of employment, conformity, predictability, and stability in relationships”).

Responses were made on a 5-point Likert scale ranging from 1 (strongly disagree) to 5 (strongly agree).

#### Extrinsic Variables

The study used eight instruments for measuring the organizational environment: Market turbulence (Narver et al., 2004), which measures the changes in the preferences and needs of customers (α = 0.69; sample item: Customers in this market are very receptive to new product ideas); Technological turbulence (Olson et al., 2005), which measures the impact of new technologies (α = 0.72; sample item: Many new product ideas have been made possible by technological advances in this industry); Competitive intensity (Jaworski and Kohli, 1993), which measures the strength of competitors (α = 0.73; sample item: There are many “promotion wars” in our industry); four scales of Competitive environment (based on the study by Porter., 2008), which measure the Power of suppliers, the Power of customers, the Threat of new entrants, and the Threat of substitute products, with each scale having one item; and finally, Speed of environmental change [based on the study by Porter (2008)], which measures how fast the seven extrinsic variables above are changing (α = 0.72; sample item: Speed of change in customers).

#### Intrinsic Variables

The organizational strategy was measured using the strategy-type instrument (Slater and Olson, 2000), which enables five different strategies to be identified: The Prospector strategy measures the behavior of being the first to market a new product or service concept; the Analyzer strategy measures the behavior of being early followers, monitoring prospector actions and customer responses to them; the Differentiating defender strategy focuses on providing different and superior levels of service and/or product quality; the Low-cost defender strategy focuses on producing goods or services as efficiently as possible and at the best price; and finally, the Reactor strategy does not appear to have a consistent product-market orientation and only responds to competitive pressures in the short term. The instrument uses one item to measure each strategy.

We also used the following eight instruments to measure organizational competencies: Responsive market orientation (MORTN; Deshpandé and Farley, 1998), which measures the activities of a company to discover and satisfy the expressed needs of the clients (α = 0.88; sample item: Our business objectives are driven primarily by customer satisfaction); Proactive market orientation (MOPRO; Narver et al., 2004), which measures the activities of the company to discover and satisfy the hidden and unconscious needs of the clients (α = 0.86; sample item: We continuously try to discover the additional needs of our customers of which they are unaware); Competitor orientation (Olson et al., 2005), which measures the organizational behaviors aimed at beating competitors (α = 0.90; sample item: We rapidly respond to competitive actions that threaten us); Speed of organizational change (based on Porter, 2008), which measures how quickly the organization adapts and is able to change based on the movements in the environment (α = 0.90; sample item: The organization adapts quickly to changes happening in the environment); and finally, four scales to measure Types of innovation (Cameron et al., 2006), radical innovation, incremental innovation, innovation in internal processes, and innovation in products and services, each of which has one item. All of the instruments used a 5-point Likert scale.

Table 1 provides a summary of the instruments along with the number of items and reliabilities.

**TABLE 1:** Summary of the instruments.

Scale	No. of items and version	Subscale (items)	Alpha C
**Scale of culture**			
OCAI. Organizational Culture Assessment Instrument (Cameron and Quinn, 2006)	English (24 items)	F1.- Clan (6 items) F2.- Adhocracy (6 items) F3.- Market (6 items) F4.- Hierarchy (6 items)	0.74 0.79 0.71 0.73
	Spanish (12 items)	F1.- *Clan* (4 items) F2.- *Mercado* (4 items) F3.- *Jerarquía* (4 items)	0.74 0.79 0.71
**Intrinsic scales (strategy and organizational variables)**			
Strategy type (Slater and Olson, 2000)	English (5 items)	F1.- Prospector (1 item) F2.- Analyzer (1 item) F3.- Low-cost defender (1 item) F4.- Differentiated defender (1 item) F5.- Reactive (1 item)	
	Spanish (5 items)	F1.- *Prospectora* (1 item) F2.- *Analizadora* (1 item) F3.- *Defensiva* low-cost (1 item) F4.- *Defensiva diferenciadora* (1 item) F5.- *Reactiva* (1 item)	
MORTN. Responsive market orientation (Deshpandé and Farley, 1998)	English (10 items)	F1.- Responsive market orientation	0.88
	Spanish (10 items)	F1.- *Orientación a Mercado* Responsive	0.88
MOPRO. Proactive market orientation (Narver et al., 2004)	English (8 items)	F1.- Proactive market orientation	0.88
	Spanish (7 items)	F1.- *Orientación a Mercado Proactiva*	0.86
Competitor orientation (Olson et al., 2005)	English (8 items)	F1.- Competitor orientation	0.90
	Spanish (8 items)	F1.- *Orientación a Competidores*	0.90
Velocidad de Cambio de la Organización	Spanish (4 items)	F1*.- Velocidad de Cambio de la Organización*	0.87
Tipo de Innovación	Spanish (4 items)	F1.- *Radical en Productos* (Radical in products) F2.- *Incremental en Productos* (Incremental in products) F3.- *Radical en Procesos* (Radical in processes) F4.- *Incremental en Procesos* (Incremental in processes)	–
**Extrinsic scales (environment variables)**			
Market turbulence (Narver et al., 2004)	English (8 items)	F1.- Market turbulence	0.69
	Spanish (7 items)	F1.- *Turbulencia de Mercado*	0.69
Technological turbulence (Olson et al., 2005)	English (8 items)	F1.- Technological turbulence	0.94
	Spanish (7 items)	F1.- *Turbulencia Tecnológica*	0.72
Competitive intensity (Jaworski and Kohli, 1993)	English (8 items)	F1.- Competitive intensity	0.81
	Spanish (6 items)	F1.- *Intensidad competitiva*	0.73
*Entorno Competitivo*	Spanish (4 items)	F1.- *Poder de negociación de proveedores* (Bargaining power of suppliers) F2.- *Poder de negociación de clientes* (Bargaining power of buyers) F3.- *Amenaza entrada nuevos competidores* (Threat of new entrants) F4.- *Amenaza de productos o servicios substitutivos* (Threat of substitute products)	**–**
*Velocidad de Evolución del Entorno*	Spanish (7 items)	F1.- *Velocidad de Evolución del Entorno* (speed of environment change)	0.72

### Procedure

Participants were obtained through non-probabilistic sampling (Hernández et al., 2000). The data were collected between December 2016 and May 2019 through an online questionnaire. The response rate was 81% for the Spanish subsample and 87% for the Peruvian subsample.

### Data Analysis

We used the IBM SPSS program (version 23.0) to carry out the stepwise multiple regressions and to calculate the reliabilities. We used multiple regressions as a way to explore, in the same study, the capacity of many variables (8 extrinsic and 13 intrinsic) to predict the presence of each type of culture. Previous studies in the literature conducted in different contexts have shown the benefits of using multiple regressions (An et al., 2011; Naranjo-Valencia et al., 2011; Hwang, 2019).

## Results

The predictive study for the Clan culture with the Spanish subsample (*n*_1_ = 362) can explain 34% of the variance with the following six predictive variables and percentages of explained variance: Speed of organizational change (22%), MORTN (7%), MOPRO (2%), Reactor strategy (1%), Analyzer strategy (1%), and Incremental innovation in internal processes (1%). The study with the Peruvian subsample (*n*_2_ = 1,317) can also explain 34% of the variance with the following seven predictive variables: MORTN (24%), Speed of change (6%), Incremental innovation in products and services (2%), Prospector strategy (1%), Analyzer strategy (1%), Reactor strategy (<1% with a negative sign), and Market turbulence (<1%).

The results show that the Clan culture, in both the Spanish and Peruvian subsamples, is mainly characterized by its capacity to change and adapt quickly (Speed of organizational change) and its ability to respond to the present desires of clients (MORTN). There is one extrinsic variable, i.e., Market turbulence, in the Peruvian subsample that has a small predictive capacity but a positive correlation. Thus, there is no indication that the Clan culture is more common in stable environments.

Tables 2, 3 show the models and coefficients of the stepwise multiple regressions.

**TABLE 2:** Summary of the models, predictive variables, and coefficients of regression analysis (stepwise method): clan culture (Spanish subsample *n*_1_ = 362).

Models and variables	Models						Coefficients				
		
	R	R^2^	R^2^ ^Adjusted^	R *^Change^*	F	Sig.	B	SE	β	*t*	Sig.
Model-1	0.47	0.22	0.22	0.22	104.56	0.000					
Model-2	0.54	0.29	0.29	0.07	73.80	0.000					
Model-3	0.56	0.31	0.31	0.02	54.52	0.000					
Model-4	0.57	0.33	0.32	0.01	44.05	0.000					
Model-5	0.59	0.34	0.33	0.01	37.18	0.000					
Model-6	0.59	0.35	0.34	0.01	32.03	0.000					
Speed of org. change							0.20	0.06	0.20	3.61	0.000
MORTN							0.08	0.03	0.17	2.86	0.004
MOPRO							0.10	0.04	0.15	2.61	0.009
Reactor strategy							–0.43	0.13	–0.15	–3.21	0.001
Analyzer strategy							0.34	0.14	0.10	2.37	0.019
Incremental innovation internal processes							0.39	0.18	0.11	2.11	0.035

**TABLE 3:** Summary of the models, predictive variables, and coefficients of regression analysis (stepwise method): clan culture (Peruvian subsample *n*_2_ = 1,317).

Models and variables	Models						Coefficients				
		
	R	R^2^	R^2 Adjusted^	R ^Change^	F	Sig.	B	SE	β	*t*	Sig.
Model-1	0.49	0.24	0.24	0.24	426.35	0.000					
Model-2	0.55	0.30	0.30	0.06	287.58	0.000					
Model-3	0.57	0.32	0.32	0.02	206.23	0.000					
Model-4	0.57	0.33	0.33	0.01	158.39	0.000					
Model-5	0.58	0.33	0.34	0.01	130.63	0.000					
Model-6	0.58	0.34	0.34	0.00	111.58	0.000					
Model-7	0.58	0.34	0.34	0.00	97.06	0.000					
MORTN							0.10	0.01	0.22	7.24	0.000
Speed of org. change							0.20	0.03	0.19	6.11	0.000
Incremental innovation products-services							0.41	0.10	0.13	4.07	0.000
Prospector strategy							0.35	0.09	0.10	3.80	0.000
Analyzer strategy							0.32	0.07	0.11	4.39	0.000
Reactor strategy							–0.25	0.07	–0.09	–3.66	0.000
Market turbulence							0.09	0.03	0.07	2.63	0.009

The predictive study for the Market culture with the Spanish subsample (*n*_1_ = 362) can explain 18% of the variance with the following three predictive variables: Competitor orientation (13%), Prospector strategy (4%), and Low-cost strategy (1%). The study with the Peruvian subsample (*n*_2_ = 1,317) can explain 32% of the variance with the following eight predictive variables: MORTN (24%), Radical innovation in products and services (3%), Low-cost strategy (2%), Incremental innovation in products and services (1%), Competitive intensity (1%), Market turbulence (1% and negative sign), Speed of organizational change (<1%), and Reactor strategy (<1%).

Unexpectedly, the results show that the predictors of Market culture are very different in the two subsamples. In the Spanish case, the Market culture is mainly characterized by the Competitor orientation and the Prospector strategy, but in the Peruvian case, it is characterized by the Responsive client orientation (MORTN). Only the Low-cost strategy is shared at a low percentage, suggesting that this culture varies greatly in its internal characteristics. Contrary to what most of the literature suggests, no extrinsic variable appears as a predictor in the Spanish subsample. In the Peruvian subsample, however, there is a 1% predictive capacity for Competitive intensity and another 1%, but with a negative sign, for Market turbulence. This suggests that Market culture could be quite common in Peru when competition increases but less common when customer preferences are changing.

Tables 4, 5 show the models and coefficients of the stepwise multiple regressions.

**TABLE 4:** Summary of the models, predictive variables, and coefficients of regression analysis (stepwise method): market culture (Spanish subsample *n*_1_ = 362).

Models and variables	Models						Coefficients				
		
	R	R^2^	R^2^ ^Adjusted^	R ^Change^	F	Sig.	B	SE	β	*t*	Sig.
Model-1	0.36	0.13	0.13	0.13	54.46	0.000					
Model-2	0.42	0.17	0.17	0.04	37.58	0.000					
Model-3	0.43	0.18	0.18	0.01	27.03	0.000					
Competitor orientation							0.10	0.02	0.24	4.53	0.000
Prospector strategy							0.76	0.16	0.25	4.66	0.000
Low-cost strategy							0.35	0.15	0.11	2.25	0.025

**TABLE 5:** Summary of the models, predictive variables, and coefficients of regression analysis (stepwise method): market culture (Peruvian subsample *n*_2_ = 1,317).

Models and variables	Models						Coefficients				
		
	R	R^2^	R^2 Adjusted^	R ^Change^	F	Sig.	B	SE	β	*t*	Sig.
Model-1	0.49	0.24	0.24	0.24	411.06	0.000					
Model-2	0.52	0.27	0.27	0.03	249.44	0.000					
Model-3	0.54	0.29	0.29	0.02	182.74	0.000					
Model-4	0.55	0.30	0.30	0.01	142.91	0.000					
Model-5	0.56	0.31	0.31	0.01	117.73	0.000					
Model-6	0.56	0.31	0.32	0.01	100.20	0.000					
Model-7	0.56	0.32	0.32	0.00	87.46	0.000					
Model-8	0.57	0.32	0.32	0.00	77.58	0.000					
MORTN							0.01	0.01	0.28	9.13	0.000
Disruptive innovation in products and services							0.30	0.09	0.12	3.47	0.001
Low-cost strategy							0.34	0.06	0.13	5.62	0.000
Incremental innovation in products and services							0.28	0.09	0.10	3.00	0.003
Competitive intensity							0.12	0.02	0.13	4.59	0.000
Market turbulence							–0.09	0.03	–0.08	–2.62	0.009
Speed of organizational change							0.08	0.03	0.09	2.73	0.006
Strategy reactor							–0.14	0.06	–0.06	–2.46	0.014

Finally, the predictive study for the Hierarchy culture with the Spanish subsample (*n*_1_ = 362) can explain 12% of the variance with the following three predictive variables: Low-cost strategy (6%), MORTN (5%), and Incremental innovation in internal processes (1%). The study with the Peruvian subsample (*n*_2_ = 1,317) can explain 23% of the variance with the following seven predictive variables: MORTN (17%), Incremental innovation in internal processes (2%), Low-cost strategy (2%), Competitor orientation (1%), Prospector strategy (1%), Threat of new entrants (<1%), and Radical innovation in internal processes (<1%).

The results show that the Hierarchy culture, in both the Spanish and Peruvian subsamples, is mainly characterized by its interest in the present needs of the clients (MORTN), the Low-cost strategy, and Incremental innovation in internal processes. There is one extrinsic variable in the Peruvian subsample with a small predictive capacity but with a positive correlation: Threat of new entrants. Thus, there is no indication that the Clan culture is more common in stable environments.

Tables 6, 7 show the models and coefficients of the stepwise multiple regressions.

**TABLE 6:** Summary of the models, predictive variables, and coefficients of regression analysis (stepwise method): hierarchy culture (Spanish subsample *n*_1_ = 362).

Models and variables	Models						Coefficients				
		
	R	R^2^	R^2 Adjusted^	R ^Change^	F	Sig.	B	SE	β	*t*	Sig.
Model-1	0.25	0.06	0.06	0.06	23.42	0.000					
Model-2	0.34	0.11	0.11	0.05	23.07	0.000					
Model-3	0.35	0.12	0.12	0.01	16.95	0.000					
Low-cost strategy							0.73	0.14	0.25	5.14	0.000
MORTN							0.07	0.02	0.18	3.23	0.001
Incremental innovation internal processes							0.34	0.16	0.11	2.07	0.039

**TABLE 7:** Summary of the models, predictive variables, and coefficients of regression analysis (stepwise method): hierarchy culture (Peruvian subsample *n*_2_ = 1,317).

Models and variables	Models						Coefficients				
		
	R	R^2^	R^2 Adjusted^	R ^Change^	F	Sig.	B	SE	β	*t*	Sig.
Model-1	0.41	0.17	0.17	0.17	263.39	0.000					
Model-2	0.44	0.20	0.19	0.02	160.47	0.000					
Model-3	0.46	0.21	0.21	0.02	118.96	0.000					
Model-4	0.47	0.22	0.22	0.01	93.94	0.000					
Model-5	0.48	0.23	0.23	0.01	76.96	0.000					
Model-6	0.48	0.23	0.23	0.00	65.04	0.000					
Model-7	0.48	0.23	0.23	0.00	56.50	0.000					
MORTN							0.07	0.01	0.20	5.25	0.000
Incremental innovation internal processes							0.31	0.10	0.11	3.19	0.001
Low-cost strategy							0.34	0.07	0.12	5.06	0.000
Competitor orientation							0.37	0.01	0.11	2.71	0.007
Prospector strategy							0.19	0.08	0.07	2.35	0.019
Threat of new competitors							0.14	0.07	0.05	2.19	0.034
Radical innovation internal processes							0.20	0.09	0.07	2.08	0.038

Tables 8–10 provide a summary of the predictive variables for each culture. They are ordered by the value of the R Change, from highest to lowest and comparing the two subsamples. The matching variables for the two subsamples in each table are highlighted in bold. This comparison of the results between the Spanish and Peruvian subsamples shows a high level of agreement between the Clan and Hierarchy cultures. However, for the Market culture, the results only have the Low-cost strategy variable in common, with less predictive capacity.

**TABLE 8:** Summary of the predictive variables for the Clan culture, sorted by the value of the R Change, from highest to lowest.

SPAIN *n*_1_ = 362	PERU *n*_2_ = 1,317
**Speed of org. change** ΔR^2^ = 0.22 (β = 0.20)	**MORTN** ΔR^2^ = 0.24 (β = 0.22)
**MORTN** ΔR^2^ = 0.07 (β = 0.17)	**Speed of org. change** ΔR2 = 0.06 (β = 0.19)
MOPRO ΔR^2^ = 0.02 (β = 0.15)	Incremental innovation in products and services ΔR2 = 0.02 (β = 0.13)
**Reactor strategy** ΔR^2^ = 0.01 (β = –0.15)	Prospector strategy ΔR^2^ = 0.01 (β = 0.10)
**Analyzer strategy** ΔR^2^ = 0.01 (β = 0.10)	**Analyzer strategy** ΔR^2^ = 0.01 (β = 0.11)
Incremental innovation in internal processes ΔR^2^ = 0.01 (β = 0.11)	**Reactor strategy** ΔR^2^ = 0.00 (β = –0.09)
	Market turbulence ΔR^2^ = 0.00 (β = 0.07)
Explained variance 34%	Explained variance 34%

*The matching variables are highlighted in bold.*

**TABLE 9:** Summary of the predictive variables for the Market culture, sorted by the value of the R Change, from highest to lowest.

SPAIN *n*_1_ = 362	PERU *n*_2_ = 1,317
Competitor orientation ΔR^2^ = 0.13 (β = 0.24)	MORTN ΔR^2^ = 0.24 (β = 0.28)
Prospector strategy ΔR^2^ = 0.04 (β = 0.25)	Radical innovation in products and services ΔR^2^ = 0.03 (β = 0.12)
**Low-cost strategy** ΔR^2^ = 0.01 (β = 0.11)	**Low-cost strategy** ΔR^2^ = 0.02 (β = 0.13)
	Incremental innovation in products and services ΔR^2^ = 0.01 (β = 0.10)
	Competitive intensity ΔR^2^ = 0.01 (β = 0.13)
	Market turbulence ΔR^2^ = 0.01 (β = –0.08)
	Speed of organizational change ΔR^2^ = 0.00 (β = 0.09)
	Reactor strategy ΔR^2^ = 0.00 (β = –0.07)
Explained variance 18%	Explained variance 32%

*The matching variables are highlighted in bold.*

**TABLE 10:** Summary of the predictive variables for the Hierarchy culture, sorted by the value of the R Change, from highest to lowest.

SPAIN *n*_1_ = 362	PERU *n*_2_ = 1,317
**Low-cost strategy** ΔR^2^ = 0.06 (β = 0.25)	**MORTN** ΔR^2^ = 0.17 (β = 0.20)
**MORTN** ΔR^2^ = 0.05 (β = 0.18)	**Incremental innovation internal processes** ΔR^2^ = 0.02 (β = 0.11)
**Incremental innovation internal processes** ΔR^2^ = 0.01 (β = 0.11)	**Low-cost strategy** ΔR^2^ = 0.02 (β = 0.12)
	Competitor orientation ΔR^2^ = 0.01 (β = 0.11)
	Prospector strategy ΔR^2^ = 0.01 (β = 0.07)
	Threat of new entrants ΔR^2^ = 0.00 (β = 0.05)
	Radical innovation internal processes ΔR^2^ = 0.00 (β = 0.07)
Explained variance 12%	Explained variance 23%

*The matching variables are highlighted in bold.*

## Discussion

### Summary and Discussion of the Results

The objective of this study was to determine which extrinsic and intrinsic variables can predict the presence of the Clan, Market, and Hierarchy organizational cultures (Cameron and Quinn, 1999). We also compared the results from a Spanish (*n*_1_ = 362) and a Peruvian subsample (*n*_2_ = 1,317). The links between the extrinsic variables and the cultures could help us to understand which cultures are better adapted to each environment. The links between the intrinsic variables and the cultures, in contrast, could give us information about which business strategies and organizational competencies are the characteristics of each culture. In addition, the differences in the results for the two subsamples could enrich our understanding of the characteristics of each culture. By gaining a better understanding of the variables of each type of organizational culture, we could better manage the suffering of employees.

Hypotheses M1, C1, and H1 focus on the relationships between cultures and the environment. Quinn and Cameron (1983); Cameron et al. (2006), and Schein (2010) have suggested that the Market culture is the most capable of surviving in an ever-changing environment, while the Clan culture and the Hierarchy culture are better adapted to stable environments. Hypothesis M1 is partially fulfilled in the Peruvian subsample. Our study demonstrates that Competitive intensity has some predictive capacity in the Peruvian subsample as far as the Market culture is concerned. This is consistent with the study by Cameron et al. (2006). Unexpectedly, we also found that Market turbulence has some predictive capacity in the Peruvian subsample, but with a negative sign. This suggests that the Market culture could be fairly common in Peru when competition is strong but less common when customer preferences are changing. Nevertheless, the two predictive capacities are small and do not appear in the Spanish subsample. Hypotheses C1 and H1 are not fulfilled. Our study found no extrinsic variable with an inverse relationship with either the Clan culture or the Hierarchy culture. Therefore, we found nothing to support the idea that the two cultures are more common in stable environments.

Hypotheses C2, M2, H2, and H3 focus on the relationships between cultures and business strategy, based on an empirical study carried out by Slater et al. (2010). Hypothesis C2 is not fulfilled. We found no support for the idea that the presence of the Clan culture can be predicted by a Differentiated defender strategy. Thus, human capital does not appear as a source of strategic differentiation. Nevertheless, our results did find some predictive capacity in the Reactor and Analyzer strategies in both Spain and Peru, as well as in the Prospector strategy in Peru. Hypothesis M2, which states that the presence of the Market culture can be predicted by the Analyzer defender strategy, is not fulfilled. Nevertheless, our results found some predictive capacity in the Low-cost strategy in both Spain and Peru, being this consistent with the findings of Nase and Arkesteijn (2018) in the global research on corporate real state. We also found some predictive capacity in the Prospector strategy in Spain and the Reactor strategy in the Peruvian subsample. This suggests that the Market culture can use a different mix of strategies to compete. Hypothesis H2, which states that the presence of the Hierarchy culture can be predicted by the Low-cost defender strategy, is fulfilled. Our results found a relevant predictive capacity in the Low-cost strategy in both the Spanish and Peruvian subsamples, supporting the idea that the Hierarchy culture uses the excellence of its processes to lower costs (Slater et al., 2010; Nase and Arkesteijn, 2018).

Hypothesis H3 states that the presence of the Hierarchy culture can be predicted by the Analyzer defender strategy. This hypothesis is not fulfilled.

Hypotheses C3, C4, M3, M4, and H4 focus on the relationships between cultures and organizational competencies, also based on the empirical research carried out by Slater et al. (2010). Hypothesis C3 states that the presence of the Clan culture can be predicted by Market orientation. Our results show that this hypothesis is fulfilled insofar as MORTN has a relevant predictive capacity in the two subsamples. This is consistent with the findings of various researchers (Jaworski and Kohli, 1993; Cameron et al., 2006; Iglesias et al., 2011). Hypothesis C4 is also fulfilled. In fact, the presence of the Clan culture can be predicted by the Speed of organizational change in both subsamples. Thus, we have support for the idea that the Clan culture is capable of changing and adapting quickly. This is consistent with the findings of Cameron et al. (2006) and Goncalves et al. (2020). Hypothesis M3 states that the presence of the Market culture can be predicted by Market orientation. This is fulfilled in the Peruvian subsample, for which we found a relevant predictive capacity in MORTN. Unexpectedly, we did not find the same link in the Spanish subsample, contrary to the theory of Cameron et al. (2006). This suggests that the Market culture may have very different competencies depending on certain circumstances that are as yet unknown. Hypothesis M4 states that the presence of the Market culture can be predicted by Competitor orientation. This is only fulfilled in the Spanish subsample, for which our results show some predictive capacity. Unexpectedly, we did not find this link in the Peruvian subsample, which again evidences high variability in Market culture competencies. Finally, Hypothesis H4 states that the presence of the Hierarchy culture can be predicted by Competitor orientation. This is fulfilled in the Peruvian subsample and shows some capacity to observe competitors in order to benchmark costs and prices. Unexpectedly, our results found a relevant predictive capacity in MORTN and Incremental innovation in internal processes in both subsamples. We also found a small predictive capacity in Radical innovation in internal processes in the Peruvian subsample. In line with the theory of Slater et al. (2010), the Hierarchy culture, therefore, seems to be oriented toward the present needs of clients and also seems capable of improving internal processes. Karakasnaki et al. (2019), in the recent research in the shipping industry, gave support to the relationship between Hierarchy culture and MORTN, finding that “the more hierarchical lines of authority and standardization of procedures are evident in an organization, the stronger the perceptions that employees care about the needs of the customers.” Both MORTN and Incremental innovation in internal processes are consistent with the Low-cost strategy, as mentioned earlier. The presence of MORTN in the Hierarchy culture could have a motivational effect on employees since satisfying customers could give meaning to their work (Jaworski and Kohli, 1993) and result in high levels of organizational commitment (Pinho et al., 2014). In the same way, participating in the processes of Incremental innovation could also be beneficial for employees.

When we compared the Spanish and Peruvian subsamples for the Clan culture, which is internally oriented (Cameron and Quinn, 1999), both showed high agreement in their main predictive variables, these being Speed of organizational change and MORTN. Both subsamples also showed a high level of agreement in the predictors of the Hierarchy culture, which are Low-cost strategy, Incremental innovation in internal processes, and MORTN. This suggests that both cultural archetypes are robust and stable. In contrast, in the Market culture, we found very different predictors in the two subsamples. This suggests that companies could develop many different configurations of business strategies and organizational competencies while maintaining the characteristic external orientation of the culture and a strong focus on results, thus having different effects on the suffering and well-being of employees.

### Limitations and Suggestions for Future Research

This study has certain limitations. We would also like to make some suggestions for future research: First, our data were obtained using the non-probabilistic sampling of Spanish and Peruvian managers. We recommend that the research should be extended to cover other employee profiles and other countries. Second, our study could not include the Adhocracy culture (Cameron and Quinn, 1999) in the regression analysis because this factor was totally excluded from the translation and adaptation of the OCAI into Spanish. Future studies should design a new scale for measuring this culture, which is characterized by its capacity for innovation. Third, unlike the Clan and Hierarchy cultures, the Market culture showed very different predictors when Spain and Peru were compared, for reasons that remain unknown to us. More research is necessary to discover how this cultural archetype acquires different intrinsic factors in different countries. Such research could include the analysis of differences in the cultural and economic contexts of each country. Fourth, complementary studies should investigate the links between the intrinsic variables of each organizational culture and suffering of employees. Finally, the complementary studies should extend the sample to specific industries. Isolating the particular characteristics of each business environment could help us to understand how a culture adapts to its own specific extrinsic factors.

## Conclusion

This study enables the following conclusions to be reached: First, the eight extrinsic variables that were analyzed showed a very less predictive capacity for the Clan, Market, and Hierarchy cultures that we have studied based on the model suggested by Cameron and Quinn (1999). This is an important contribution to this study because, even though there is an agreement in academia that the business environment has a decisive influence on the survival of a company, this study found no pattern to suggest that specific cultures are more likely to be found in particular environments. In conclusion, in all the environments, the three types of culture could influence suffering at work. Second, 11 of the intrinsic variables that were researched have a relevant predictive capacity for the Clan and Hierarchy cultures in both the Spanish and Peruvian subsamples. These results supply valuable information about the business strategy and organizational competencies of these three cultural archetypes, providing stronger empirical support for previous research. Specifically, the main characteristics found in the Clan culture are MORTN and Speed of organizational change. This is, therefore, a culture that is committed to its customers and can change and adapt quickly. By giving meaning to the work, MORTN could create synergies with the human approach that characterizes the Clan culture, helping to reduce suffering at work. The main characteristics of the Hierarchy culture, in contrast, are Low-cost strategy, Incremental innovation in internal processes, and MORTN. This is, therefore, a culture that competes by lowering its costs and prices, constantly improving its internal processes to achieve this, and is also committed to its customers. MORTN and participating in the processes of Incremental innovation could be beneficial in reducing suffering at work by giving meaning to work and allowing employees to use their ingenuity. Finally, 10 of the intrinsic variables have a relevant predictive capacity for the Market culture; however, unexpectedly, these are very different in the two subsamples. The main characteristics found in the Spanish subsample are the Competitor orientation and the Prospector strategy, whereas, in the Peruvian subsample, they are MORTN and Radical innovation in products and services. Only the Low-cost strategy is shared by the two subsamples. This is another important contribution of this study and suggests that the Market culture may have different internal configurations while maintaining its characteristically strong focus on results and aggressive competitiveness, thus potentially having very different effects on suffering at work.

In conclusion, there is a link between organizational culture and suffering at work, and this study has found important characteristics of each type of organizational culture that could be useful to better manage the suffering of employees.

## Data Availability Statement

The raw data supporting the conclusions of this article will be made available by the authors, without undue reservation.

## Ethics Statement

Ethical review and approval was not required for the study on human participants in accordance with the local legislation and institutional requirements. The patients/participants provided their written informed consent to participate in this study.

## Author Contributions

JA-S, MB-C, and EA-T contributed to the conceptualization. JA-S, M-JS-F, and EA-T contributed to the methodology. JA-S, MB-C, M-JS-F, and EA-T contributed to the writing—original draft preparation. All authors have read and agreed to the published version of the manuscript.

## Conflict of Interest

The authors declare that the research was conducted in the absence of any commercial or financial relationships that could be construed as a potential conflict of interest.

## Publisher’s Note

All claims expressed in this article are solely those of the authors and do not necessarily represent those of their affiliated organizations, or those of the publisher, the editors and the reviewers. Any product that may be evaluated in this article, or claim that may be made by its manufacturer, is not guaranteed or endorsed by the publisher.
